# S117. MODELLING THE RELATIONSHIP BETWEEN INSIGHT, PSYCHOPATHOLOGY AND GENDER IN SCHIZOPHRENIA USING STRUCTURAL EQUATIONS

**DOI:** 10.1093/schbul/sby018.904

**Published:** 2018-04-01

**Authors:** Jesus Cobo, Javier Labad, Esther Pousa, Lourdes Nieto, Susana Ochoa, Judith Usall, Iris Baños, Beatriz González, Carmina Massons, Isabel Ruiz, Ada Ruiz

**Affiliations:** 1 Corporació Sanitària Parc Taulí; 2 Corporació Sanitària Parc Taulí, Hospital Universitari – UAB - CIBERSAM; 3 Institut de Neuropsiquiatria i Addiccions, Hospital del Mar; 4 Instituto Nacional de Psiquiatría Ramón de la Fuente Muñiz; 5 Parc Sanitari San Joan de Dèu - CIBERSAM; 6 Hospital Benito Menni; 7 Corporació Sanitària Parc Taulí, Hospital Universitari; 8 Universitat Autònoma de Barcelona

## Abstract

**Background:**

Studies about the problem of the lack of awareness of the illness in psychosis have a long trajectory of research (Amador and David, 2004). Previous reported articles exert their insidious negative effects in the evolution and managing of the illness both by the patients and professionals. Lack of insight have been related to a generally poor prognosis of schizophrenia, predisposing to non-adherence with antipsychotic and other negatives influences on outcomes. In the last years, previous analysis from the Insight Barcelona Research Group tried to develop a deeper view of insight dimensions in schizophrenia (Pousa et al., 2017).

The impact of gender in schizophrenia and first-episode psychosis has been studied extensively in recent decades (Riecher-Rössler and Häfner, 2000; Ochoa et al., 2011). Previous studies about the role of gender in the deficit of insight in psychosis showed inconclusive results. These contradictory findings may be related to differences in the study populations as well as to the use of different instruments to assess insight and other related variables. In these sense, our group presented a previous analysis focused on insight and gender (Cobo et al., 2016) into specific psychotic symptoms. Using the Spanish complete version of the Scale of Unawareness of Mental Disorder - SUMD (Ruiz et al., 2008) and the Five-PANSS Lidenmayer’s Factors (1995) - Positive, Negative, Cognitive, Depressive and Excitement. No gender differences in the three main dimensions of insight in psychosis were found, neither in awareness and attribution of symptoms when assessed globally. However, gender differences appear in awareness and attribution of particular symptoms when assessed separately, with women showing higher levels of unawareness and misattribution than men. On the other hand, a different pattern of clinical, sociodemographic and functional variables seem to affect insight in men and women differently.

**Aim:**

The aim of this study was to modelling the influence of psychopathological factors in the deficit of insight in psychosis, taking in account the difference of gender as a relevant variable.

**Methods:**

A multicenter sample of 305 patients with schizophrenia who agreed to participate was evaluated in four centers of the metropolitan area of Barcelona (Catalonia). Psychopathological assessment was performed using the Five-PANSS Wallwork’s Factors (2012). Insight and its dimensions were assessed by means of the SUMD. Structural Equation Models (SEM) was used to fix the model in the total sample and by gender.

**Results:**

SEM models for the sample showed a moderate fix capacity. Insight SUMD Dimensions models in the schizophrenia sub-sample were related significantly to Positive, Excited and Depressed PANSS Wallwork’s Factors. Higher scores on Positive and Excited PANSS Wallwork’s Factors reduce de insight scores. Conversely, higher scores in Depressed PANSS Wallwork Factor were associated to greater awareness. In women affected of schizophrenia, SEM models fixed poorly and the lack of association for any isolated PANSS factor, probably due to the size of the subsample.

**Discussion:**

There is a lack of studies in the area. In the previous SEM model of Xavier et al. (2017), disorganized symptoms had the strongest effect on insight. In addition, both in our study and in the study of Xavier et al. (2017), negative symptoms have no significative effect on either illness insight or treatment insight. In our opinion, the possible explanation of the principal differences between the studies is related to the instruments utilized and the sample characteristics. Our data also support gender differences in the influence of psychopatology factors on the insight dimensions.

